# Supplementary material for: OpenMM 7: Rapid development of high performance algorithms for molecular dynamics
Source: PLoS Comput Biol. 2017 Jul 26;13(7):e1005659. doi: 10.1371/journal.pcbi.1005659 (PMC5549999; doi:10.1371/journal.pcbi.1005659)
Supplement: S1 Source Code — (ZIP) [file pcbi.1005659.s002.zip › OpenMM-7.0.1-Source/docs/api-c++/generated/OpenMM.Discrete2DFunction.html]

Discrete2DFunction — OpenMM @OPENMM\_MAJOR\_VERSION@.@OPENMM\_MINOR\_VERSION@ documentation


# Discrete2DFunction¶

*class* `OpenMM::``Discrete2DFunction`¶
:   This is a `TabulatedFunction` that computes a discrete two dimensional function f(x,y). To evaluate it, x and y are each rounded to the nearest integer and the table element with those indices is returned. If either index is outside the range [0, size), the result is undefined.

    Methods

    |  |  |
    | --- | --- |
    | `Discrete2DFunction` | Create a `Discrete2DFunction` f(x,y) based on a set of tabulated values. |
    | `getFunctionParameters` | Get the parameters for the tabulated function. |
    | `setFunctionParameters` | Set the parameters for the tabulated function. |
    | `Copy` | Create a deep copy of the tabulated function |

    `Discrete2DFunction`(int *xsize*, int *ysize*, *const* std::vector<double> &*values*)¶
    :   Create a `Discrete2DFunction` f(x,y) based on a set of tabulated values.

        |  |  |
        | --- | --- |
        | Parameters: |  |

        - **xsize** – the number of table elements along the x direction
        - **ysize** – the number of table elements along the y direction
        - **values** – the tabulated values of the function f(x,y), ordered so that values[i+xsize\*j] = f(i,j). This must be of length xsize\*ysize.

    void `getFunctionParameters`(int &*xsize*, int &*ysize*, std::vector<double> &*values*) *const*¶
    :   Get the parameters for the tabulated function.

        |  |  |
        | --- | --- |
        | Parameters: |  |

        - **xsize** – [out] the number of table elements along the x direction
        - **ysize** – [out] the number of table elements along the y direction
        - **values** – [out] the tabulated values of the function f(x,y), ordered so that values[i+xsize\*j] = f(i,j). This must be of length xsize\*ysize.

    void `setFunctionParameters`(int *xsize*, int *ysize*, *const* std::vector<double> &*values*)¶
    :   Set the parameters for the tabulated function.

        |  |  |
        | --- | --- |
        | Parameters: |  |

        - **xsize** – the number of table elements along the x direction
        - **ysize** – the number of table elements along the y direction
        - **values** – the tabulated values of the function f(x,y), ordered so that values[i+xsize\*j] = f(i,j). This must be of length xsize\*ysize.

    Discrete2DFunction \*`Copy`() *const*¶
    :   Create a deep copy of the tabulated function

High performance molecular simulation on GPUs


### Navigation

- Core Objects
- Forces
- Integrators
- Extras

©2015, Stanford University and the Authors.
|
Powered by Sphinx 1.3.1
& Alabaster 0.7.7
|
Page source
